# Cost effectiveness of pre-hospital extracorporeal cardiopulmonary resuscitation for out-of-hospital cardiac arrest – An analysis of the PRECARE study

**DOI:** 10.1016/j.resplu.2026.101242

**Published:** 2026-01-22

**Authors:** Fredrick Zmudzki, Brian Burns, Natalie Kruit, Changle Song, Emily Moylan, Paul Forrest, Miles Greenberg, Thomas Evens, Anthony Keech, Mark Dennis

**Affiliations:** a Époque Consulting, Sydney, Australia; b Social Policy Research Centre, University of New South Wales, Sydney, NSW, Australia; c Care and Public Health Research Institute (CAPHRI), Maastricht University, Maastricht, the Netherlands; d Faculty of Medicine and Health, University of Sydney, Sydney, Australia; e Aeromedical Operations, New South Wales Ambulance, Sydney, Australia; f Faculty of Engineering, Sydney, Australia; g Royal Prince Alfred Hospital, Sydney, Australia; h NSW Ambulance, Sydney, Australia

**Keywords:** Extracorporeal membrane oxygenation, ECMO, Cardiopulmonary resuscitation, CPR, Extracorporeal membrane oxygenation cardiopulmonary resuscitation, ECPR, Prehospital ECPR, Extracorporeal cardiopulmonary resuscitation, OHCA, Out of hospital cardiac arrest

## Abstract

**Background:**

While the use of pre-hospital extracorporeal cardiopulmonary resuscitation for refractory out of hospital cardiac arrest is increasing, there is little data on whether it is cost-effective. This study investigated its cost-effectiveness of based on our current study data.

**Methods:**

Using data from the PRECARE trial, the New South Wales Ambulance Cardiac Arrest Registry (CAR) and in-hospital costings, we performed a cost effectiveness analysis of pre-hospital extracorporeal cardiopulmonary resuscitation (ECPR). A Markov model was used to integrate PRECARE service costs and patient outcomes, team time allocation to ECPR, patient volume, organ donation and alternate pre-hospital delivery strategies. Bridging formulae were used with ECPR survivor Cerebral Performance Category scores to estimate Quality Adjusted Life Years and Incremental Cost Effectiveness Ratios. Probabilistic sensitivity analysis was undertaken to assess the joint uncertainty in model parameters.

**Results:**

Sixteen patients were analysed (mean age 52 ± 10 years). Five patients (31%) survived to hospital discharge; all with a cerebral performance category score of 1 or 2. Three (60%) of survivors returned to work during the study period. There was one organ donor. The total cost per patient was $94,460 (±$103,455), with a base-case incremental cost-effectiveness ratio of AUD 34,000 per quality adjusted life year (assuming 100 patients per year, and ECPR cases occupying 15% of the PH-ECPR team’s time). If the PRECARE team were exclusively dedicated to ECPR cases, the cost per quality adjusted life year would increase to $95,000.

**Conclusion:**

PH-ECPR in Sydney is likely to be cost-effective, assuming a 15% allocation of prehospital team time to ECPR. Survival rate, organ donation and the team’s ability to perform other clinical tasks when not performing ECPR are key factors influencing cost effectiveness. A PH team exclusively dedicated to ECPR is much less cost-effective.

## Introduction

The use of extracorporeal cardiopulmonary resuscitation (ECPR) in refractory out of hospital cardiac arrest (OHCA) is increasing, with 30–40% survival in well organised systems^1^ that can ideally initiate support within 60 min. However, this is often not feasible in many cases owing to geographical and time constraints, thereby limiting patient access to timely ECPR to a small catchment area around ECPR-capable hospitals.^2^ By contrast, initiating ECPR at the scene of cardiac arrest (pre-hospital ECPR or PH-ECPR) may significantly increase patient access, reduce low-flow times and improve survival.^3^

Despite increasing utilisation, there is limited data on the overall cost-effectiveness of ECPR^4^, ^5^ and results have been conflicting.^4^, ^6^, ^7^ Moreover, the available data is predominantly observational, has marked variation in methodological approach^8^ and in organ donation effect reporting. There exists even less such data on the cost effectiveness specifically on PH-ECPR. Initial studies focussing on PH-ECPR suggest that it may be cost-effective,^9^, ^10^, ^11^ these investigations to date have been modelling studies and not yet based on trial or service data.

Therefore, we conducted the first cost-effectiveness analysis on real world trial data from the PRECARE PH-ECPR trial in Sydney, Australia. All costs to establish, staff, implement, team build, train and educate as well as patients costs and outcome data were included. Organ donation and its effect on cost effectiveness were also reported.

## Methods

### Patient study group setting

This health economic analysis is based on data from the ongoing PRECARE Study from August 2023 to April 2025; a prospective, single-arm feasibility trial where the protocol and results thus far have been reported in detail previously.^12^ In brief, the pre-hospital ECPR team consisted of two specialist pre-hospital physicians (cannulation team) and one critical care paramedic (ECMO pump management), who had undergone a specific training programme and accreditation process for the study.^13^ This team responded together in a single rapid response road vehicle within the greater Sydney Metropolitan area. All receiving hospitals were ECMO-capable centres, which already offer in-house ECPR for OHCA. Potential ECPR cases were identified by a dedicated OHCA clinician located in the NSW Ambulance Control Centre. The team was dispatched to any patient under the age of 70yrs who had a witnessed collapse. En route to the arrest, an initial situational report from the crew on scene was provided and the pre-hospital team were either stood down or continued to the cardiac arrest thereafter. On arrival to the scene, advanced cardiac life support was continued and the PH-ECPR inclusion criteria assessed which was: age 18–70 years, witnessed cardiac arrest, bystander CPR commenced in less than 5 min, initial rhythm VT/VF on EMS arrival, PEA with signs of life, time to commence cannulation <45 min from time of collapse and at least 15 min of resuscitation. If meeting inclusion criteria and not meeting exclusion criteria (i.e. ROSC obtained, terminal disease present, significant co-morbidities with severe functional impairment) ECPR was commenced and the patient was transferred to one of three ECMO centres for further management.

The Sydney Local Health District Human Research Ethics Committee approved the study (HREC Project Number: X23-0150 & 2023/ETH00767) and the trial was registered with the Australia and New Zealand Clinical Trials Registry (ACTRN12625000318482).

### PH-ECPR base case service model and scenarios

Primary base case cost effectiveness modelling was performed using annualised data from the current PRECARE PH-ECPR service, assuming 24/7 operation. Supplementary scenarios were performed to assess alternative service models including: a 12 h (9:00 am to 9:00 pm) 7 days per week service; a hospital based mobile team dispatched from an established ECMO centre to provide PH-ECPR for 12 and 24 h, 7 day per week service; and the existing in-hospital based ECPR for OHCA service.^9^ Further specification of base case and alternative scenarios are provided in the Supplementary Appendix.

### Pre-hospital ECPR costs

Pre-hospital costing was based on annualised PRECARE service data. Staffing of the PH-ECPR team comprises of two senior aeromedical doctors and one critical care paramedic (CCP). Costing includes ECPR specialist training for doctors and paramedics and a dedicated car for the pre-hospital team. A dedicated specialist out of hospital cardiac arrest (OHCA) paramedic dispatcher for case identification, a paramedic educator and a senior physician ECMO clinical lead are also costed in the base case. As these roles are not anticipated to be included in any service post-trial, additional models excluding these costs are provided in the Supplementary material. Service costs were calculated for a 24 h, 7-day-per-week service, including estimated shift penalties and on-costs for paramedics and doctors as per state awards. The trial service responded from an optimal location determined by geospatial analysis to access a majority of the Sydney metropolitan area within 45 min by road ambulance.^3^

### Patient costs

Patient “costs” were calculated as the total episode of care expenses from cardiac arrest to hospital discharge or death, covering all care elements including ambulance, medical, nursing and allied health staffing costs, pharmacological therapy, diagnostic and therapeutic procedures, consumables, clinical complications, blood products and hospital overheads including intensive care and ward lengths of stay (LOS). Unit costs were sourced from the ECMO Australian Refined Diagnosis Related Group (ARDRG), the Australian Medicare Benefits Schedule (MBS) and Pharmaceutical Benefits Scheme (PBS) consistent with standards for health economic evaluation in Australia^14^, ^15^ and previous published hospital based ECPR studies.^4^ Additional costs not included in the ECMO DRG, such as clinical procedures, surgeries, and complications, were reviewed and costed individually for each patient.

ECMO DRG figures were previously cross validated with disaggregated individual component costs to ensure accuracy of ECMO DRG cost weights.^4^ The current analysis uses ECMO AR-DRG version 11.0 2022–23, based on calculated average cost per day of $8893. A half day adjustment was made to avoid double counting as initial patient assessment and cannulation is provided and costed in the prehospital service. Consistent with the conservative approach to costing sources ICU costs are based on Australian metropolitan ICUs at $6547 per day.^16^, ^17^ Costs incurred after hospital discharge were not evaluated. All costs are presented in 2025 Australian dollars.

### Patient outcomes

Cerebral performance category (CPC) scores were recorded at discharge for all patients and bridging formulae were used to estimate Quality Adjusted Life Years (QALYs).^18^ The primary outcome was cost per QALY. Secondary outcomes included cost per survivor, QALY gained and incremental cost effectiveness ratios (ICERs). Supplementary modelling scenarios were completed as described below.

#### Statistical analysis

Categorical variables were reported as counts and percentages, while continuous variables were summarised using n, mean, and standard deviation. Due to the right-skewed nature of patient-level acute care costs, we present mean costs (with bootstrap uncertainty) for economic analysis and budget planning, as recommended for presentation of skewed health-care data^19^, ^20^ with medians and interquartile ranges, for reference, provided in Supplementary Appendices, Tables S1a and S1b. All analyses were conducted using Microsoft Office 365 for Business and STATA v16.1 (StataCorp LP, College Station, Texas, USA).

#### Modelling

**Fig. 1:**
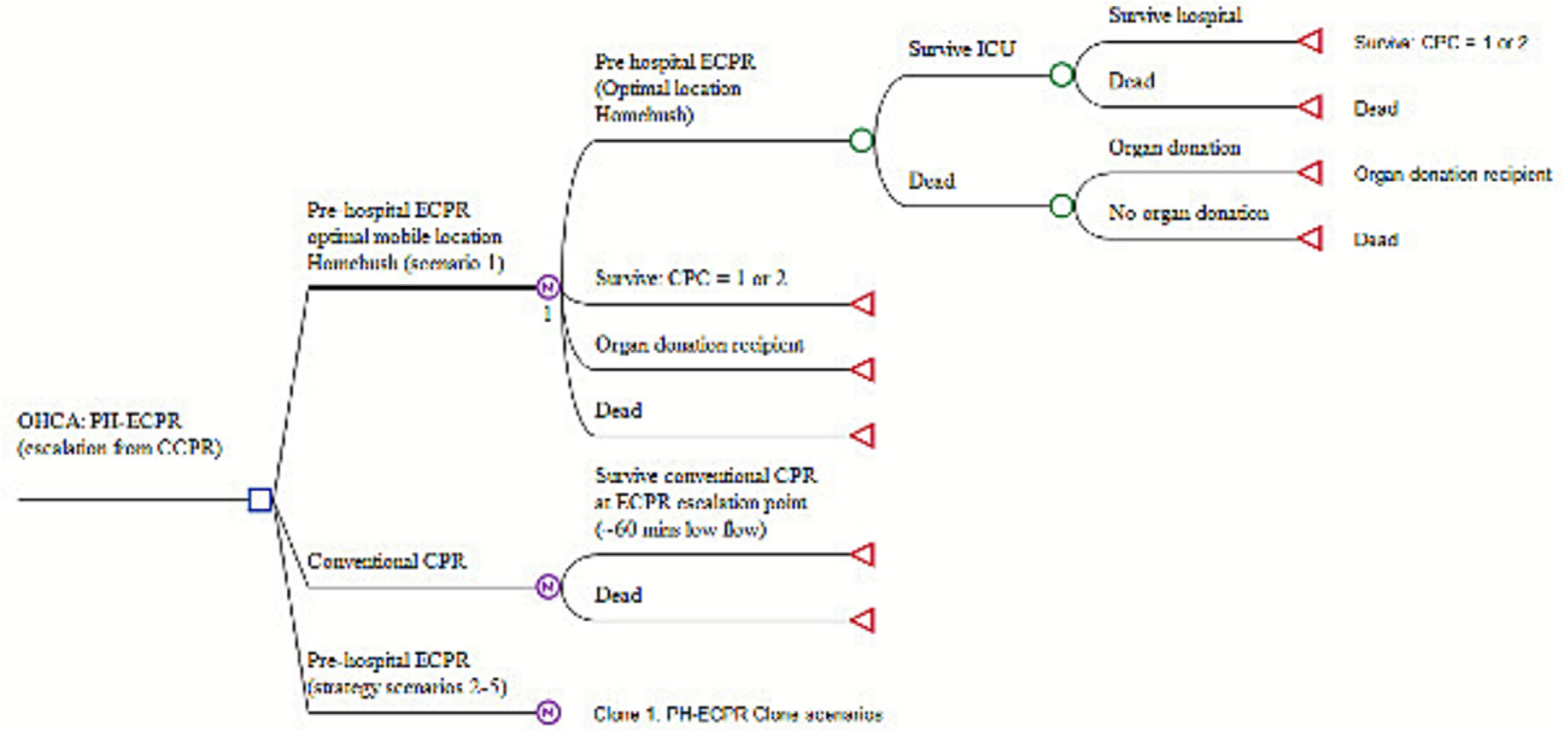
**PH-ECPR Markov model structure and patient progression pathways**. *Note:* Basic Markov model. Further details of the complete Markov model framework provided in Supplementary material A.

The base‑case compared escalation to PH‑ECPR with continued CCPR at the ECPR escalation decision point, reflecting that ECPR is a continuation of CCPR rather than an alternative treatment, consistent with our prior economic model.^4^ Our previously developed Markov model was extended^9^ to include PH-ECPR trial staffing, as well as patient cost and outcome data – Fig. 1 (full model provided in Supplementary Appendix A, Fig. S1 and Supplementary Appendix B, Table S2). Uncertainty was assessed via non-parametric bootstrapping for probabilistic sensitivity analysis (PSA). Cost effectiveness was shown as cost effectiveness acceptability curves (CEACs) reflecting the likelihood that ECPR is cost effective at different willingness-to-pay thresholds. Implementation costs were calculated from cardiac arrest to hospital discharge from an Australian health system perspective and adjusted to 2025 AUD, while all future modelling costs and QALYS beyond discharge (including organ donation recipient cost offsets for dialysis avoided minus transplant maintenance costs) were discounted at 3.5% per annum a 10‑year model horizon. CPC scores at discharge were converted to utilities using conservative assumptions from our previous ECPR economic model,^4^ reflecting the limitations of CPC to utility conversion. CPC 1 corresponds to utilities above 0.8, while CPC 2 is assigned a value near 0.4, consistent with reported CPC – health utility index relationships.^18^ The Markov model design framework were developed using TreeAge Pro 2025 r2.0. All modelling was completed in line with the Health Economic Evaluation Reporting Standards CHEERS checklist.^20^

### Survival outcomes and health-related quality of life (HRQoL)

The PH-ECPR survival rate is based on current PRECARE trial data, cross validated with previously reported ECPR studies.^3^, ^21^, ^22^, ^23^, ^24^, ^25^ The base case model assumed a mean utility of 0.85 with a standard deviation of 0.05 to provide an approximate spread above the expected minimum of 0.8.^18^ Long term ECPR follow up indicates a small percentage of survivors experience poor neurological outcomes (2.9%) of CPC 3,^26^ with some patients improving from CPC 2 to CPC 1 at 6 months and most CPC 3 and 4 patients not surviving beyond this 6 month point, therefore the implications for ongoing care and cost effectiveness is likely limited.^26^

### Base case PH-ECPR team allocation and patient numbers

In addition to ECPR cases, the PH-ECPR team is also tasked with other high acuity non ECPR patient responses e.g. trauma, non ECPR eligible OHCA cases and severe life-threatening medical emergencies. That is, the team is not only attending ECPR cases and therefore, the cost of the PH-ECPR team was apportioned according to PH-ECPR utilisation. From experience each ECPR case utilises the team for approximately 4 h. Our previous registry identified approximately 100 ECPR cases a year^27^ (assuming a 24/7 service); therefore ECPR cases are likely to constitute approximately 400 h of work, i.e. approximately 5% of total hours in a year. Additional training and other equipment maintenance is estimated to increase ECPR dedicated work to 500 h (or 6% of total year). For the base case, a 15% team cost allocation to ECPR was used to maintain a conservative approach (more cost attributed to ECPR). This was completed to ensure the approach was as conservative as possible, to avoid any potential view of bias towards PH-ECPR cost effectiveness, and to ensure any additional unmeasured or unknown costs were catered for. A post hoc sensitivity analysis assuming 5% cost allocation to ECPR was also completed and a lower 50 patients per year scenario is included to examine potential ECPR case number variation.

### Organ donation and patient pathways

The impact of patient organ donation was assessed using trial data. The study conservatively only included kidney donation with previously reported average of 1.5 recipient transplants per donor and a one-year graft survival rate of 94.1%.^28^ Organ donation improves PH-ECPR cost effectiveness by reducing recipients’ dialysis needs, which offsets transplant costs in the first year and leads to sustained ongoing dialysis cost savings, as well as quality of life improvements with recipients reporting an increase of 0.12 QALYs per year.^29^ Only kidney donation was assessed as to ensure the most conservative approach to modelling of ECPR cost effectiveness and benefits. Moreover, robust data on the benefit of kidney donation is validated and available.^30^ A post-hoc organ donation sensitivity analysis assuming no organ donation to articulate the effect of organ donation was completed.

## Results

From August 2023 to April 2025, sixteen PH-ECPR patients (mean age of 52 ± 10 years); 15 (94%) male, were included. Key patient characteristics and major interventions are provided in Table 1. Five patients (31%) survived to hospital discharge, all five (100%) survivors recorded a CPC score of 1, one case had initial CPC of 2 which improved to CPC1 at 3-month follow up. Three (60%) survivors returned or were planning to return to work during the study period (mean age 47 years). Of the remaining two survivors, one was already retired from work, and one returned home overseas. One patient (6.3%) consented to organ donation and supported successful kidney transplantation.

**Table 1:** Demographics and baseline data.

	**Baseline data**				**Intervention and complication**		
		**(%)**	**SD**	**Range**		**Patients (*n*)**	**(%)**
Mean age (years)	52		10	36–70	Coronary angiography	16	100
Male (*n*)	15	94			Coronary stenting	11	69
Mean ECMO* duration (days)	3		3	1–11	Required red blood cell transfusion	7	44
**Initial rhythm**					Renal replacement required	6	38
Ventricular fibrillation/tachycardia	15	94			Required fresh frozen plasma	5	31
Pulseless electrical activity	1	6			Major bleeding event	5	31
Paramedic defibrillations	5		3	1–10	Intra-aortic balloon pump	4	25
Witnessed	16	100			Fractured ribs	3	19
					Pulmonary haemorrhage	3	19
**Key arrest times (min)**					Pericardial effusion	2	13
Time from dispatch to departure	1		1	0–3	Cannular vascular complication	2	13
Dispatch to arrival on scene	20		6	9–35	Thrombotic event	2	13
Arrest to commence cannulation	31		11	17–62	Infection	2	13
ECPR$ team arrival to ECMO flow	27		9	16–53			
Total low flow time	38		11	10–56			
**Patient outcomes**							
Survived hospital discharge	5	31					
CPC# 1 or 2 (5/5)	5	100					
Returned to work (3/5)	3	60					
Organ donation	1	6					

* Extracorporeal membrane oxygenation.

$ Extracorporeal cardiopulmonary resuscitation.

# Cerebral performance category.

### Cost analysis

The annualised PRECARE pre-hospital team staffing and training costs are reported in Table 2. Assuming 100 patients per year at 100% of the team’s time being spent on ECPR (i.e. the team only attends ECPR cases), the pre-hospital cost per patient is $57,383. When 15% of total 24/7 service time is apportioned to ECPR the cost per patient of the PH-ECPR team is $8607.

**Table 2:** Prehospital ECPR annual service cost and PH-ECPR team cost per patient.

**Clinical role**	**Hours per week**	**Cost per week**	**Cost per annum**
Senior Staff Specialist 1 Cannulator	168	$37,338	$1,941,576
Senior Staff Specialist 2 Cannulator	168	$37,338	$1,941,576
Senior Staff Specialist – ECPR Clinical Lead	38	$8446	$439,166
Critical Care Paramedic – Perfusionist	168	$9878	$513,677
Paramedic Specialist – OHCA Clinician (Dispatch)	168	$9475	$492,710
Paramedic Educator – Perfusionist	38	$2982	$155,076
Total wages for 24 h 7 days per week		$105,457	$5,483,782

Staff Specialist training			$120,015
Porcine lab coordination			$9000

**Paramedic training (4 weeks)**			
Senior Staff Specialist – ECPR Clinical Lead			$33,782
Critical Care Paramedic – Perfusionist			$17,875
Paramedic Educator			$23,858
PH-ECPR car lease and running cost per annum			$50,000
PH-ECPR total annual service cost			$5,738,312
Average cost per patient (assumed 100 patients/year)			$57,383

**Estimated average cost per patient by assumed % PH-ECPR activity**			
50% PH-ECPR (50% other advanced critical care interventions)			$28,692
25% PH-ECPR (75% other advanced critical care interventions)			$14,346
15% PH-ECPR (85% other advanced critical care interventions)			$8607

*Source:* NSW Ambulance, PH-ECPR PRECARE trial annualised team costs. Average cost per patient assumes 100 PH-ECPR patients per annum. PH-ECPR base case assumes 15% team allocation for ECPR patient responses, turn backs and related activity. Figures reported in 2025 AUD.

Following a PH-ECPR case, average hospital care costs were $86,167 per patient, cost of cardiac interventions $5783 and complications $2510, resulting in a total cost per PH-ECPR patient of $94,460 ± $103,455, see Table 3. Median and interquartile range for survivors, non‑survivors and the total cohort are presented in Supplementary Appendix B, Tables S1a and S1b). If there were more than 100 PH-ECPR patients annually the average cost per patient would decrease as team staffing, training, and fixed service expenses are spread over a larger patient group each year. The mean total cost per PH-ECPR survivor was $153,423 ± 101,846. The mean total ECMO related costs for survivors and non-survivors was $23,656 ± 11,365 and $26,357 ± 35,342 respectively. Survivors had a mean intensive care unit (ICU) length of stay (LOS) of 13.7 ± 9.9 days at a mean cost of $89,825 ± 65,075 versus 3.8 ± 4.8 at $24,819 ± 31,522 for non-survivors. Following ICU discharge; survivors had a mean LOS in hospital of 23.8 ± 18.2 days in hospital at an estimated cost of $22,124 ± 16,849).

**Table 3:** Prehospital ECPR resource use and cost summary.

**Cost item**	**Survivors**					**Non-Survivors**						**Total**				
	***n***	**Mean LOS**	**SD LOS**	**Mean Cost (AUD)**	**SD (AUD)**	***n***		**Mean LOS**	**SD LOS**	**Mean Cost (AUD)**	**SD (AUD)**	***n***	**Mean LOS**	**SD LOS**	**Mean Cost (AUD)**	**SD (AUD)**
PH-ECPR				8607						8607					8607	
ECMO duration	5	3	1.3	23,656	11,365		11	3.5	4.0	26,357	35,342	16	3.4	3.3	25,513	29,476
ICU length of stay	5	13.7	9.9	89,825	65,075		11	3.8	4.8	24,819	31,522	16	6.9	8.0	45,133	52,537
Hospital length of stay	5	23.8	18.2	22,124	16,849		0	−	−	−	−	5	7.5	14.8	6914	13,707
Total ECMO and Hospital	5			144,212	93,289		11			59,783	66,863	16			86,167	95,719

Cardiac interventions				5090	2642					6099	4661				5783	4071
Complications				4121	5915					1777	2066				2510	3666

**Total cost**	**5**			**153,423**	**101,846**		**11**			**67,658**	**73,590**	**16**			**94,460**	**103,455**

#### Modelling

**Fig. 2:**
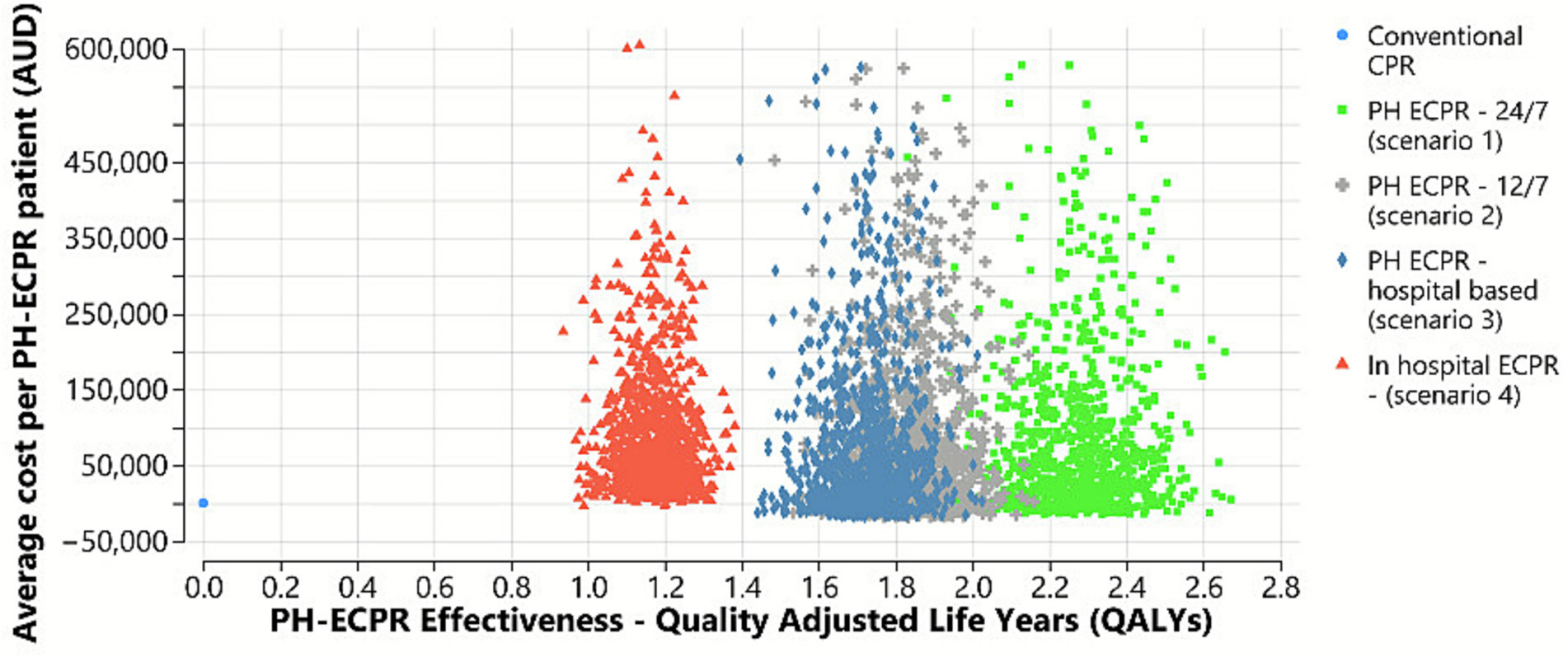
**PH-ECPR base case and alternative scenario cost effectiveness**. Bootstrapped point estimates in the scatterplot show significant variation in extrapolated ECPR cost pathways reflecting the small PRECARE patient group (*n* = 16).

The base case average incremental cost for all PH-ECPR patients (survivors and non survivors) was $77,469 with an estimated increase of 2.3 QALYs – Fig. 2 (additional cost‑effectiveness plane provided in Supplementary Appendix C, Fig. S2). The 12-h scenario (AUD 73,829) and hospital-based PH-ECPR (AUD 74,328) scenario give similar average cost per patient but with lower QALY’s of 1.8 and 1.7 respectively reflecting decreased population reach as the hospital-based team leaves from a non-optimal location (less population accessible). Substantial cost variability on bootstrapped modelling (wide range of coloured dots) is owing to the small sample size.

### Cost effectiveness acceptability

**Fig. 3:**
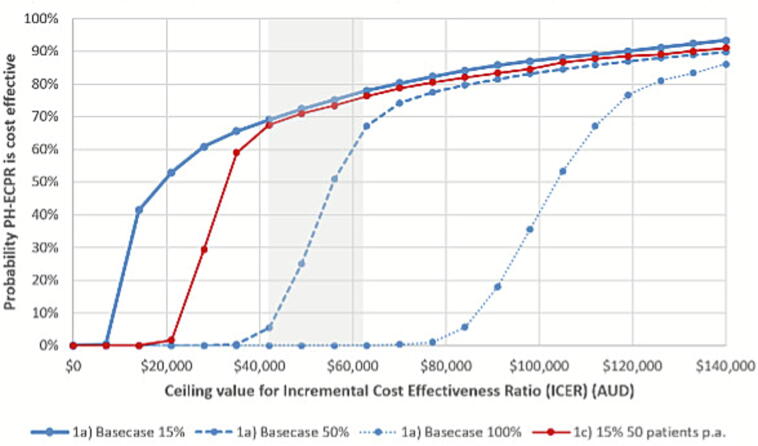
**PH-ECPR base case and alternative scenario cost effectiveness**. *Source:* PH-ECPR base case assumes 15% team allocation for ECPR patient responses, turn backs and related activity. Figures reported in 2025 AUD. Notes: The cost effectiveness thresholds are indicated by the shaded segment based on United Kingdom NICE guidance of GBP 20,000–30,000 converted at 2.09 AUD = AUD 41,800–62,700.

Cost-effectiveness acceptability curves (CEACs) in Fig. 3 show the probability that PH-ECPR is cost effective at various willingness-to-pay thresholds compared to the UK NICE reference range (shaded segment).^31^ By comparison the American College of Cardiology/American Heart Association Joint Committee defines USD 120,000 (∼180,000 AUD) per QALY as a benchmark for high value cardiac care such as PH-ECPR, around three times the Australian level.^32^ The base case (solid blue line – Fig. 3) with 15% ECPR allocation reports an incremental cost effectiveness ratio (ICER) of approximately AUD 34,000 per QALY. Owing to the small sample size, confidence in this estimate remains limited, reflected by the flattening curves at 70 to 80%. Reducing the cost allocation to ECPR to 5%, the average cost per PH-ECPR patient is $2869 and the estimated cost per QALY reduces to $31,638 (Supplementary Appendix C).

The additional blue curves in Fig. 3 represent the total ECPR team cost under scenarios of 50% ECPR utilisation at approximately $42,000 per QALY (dashed blue) and full 100% allocation around $95,000 per QALY (dotted blue) – a higher team proportion for PH-ECPR patient outcomes is associated with lower cost-effectiveness. An alternative staffing model with ECPR clinical lead provided by a physician on duty and excluding a dedicated paramedic dispatcher and educator was also evaluated, indicating the potential for improved cost-effectiveness at each level of team ECPR utilisation if such a reduction is feasible (Supplementary Appendices D and E). For scenarios involving only 50 PH-ECPR eligible patients per year, cost-effectiveness is reduced to approximately $38,000 per QALY due to an increased average cost per patient (solid red line).

### Organ donation sensitivity analysis

On removal of organ donation related benefits, the PH-ECPR costs increased to $45,025 /QALY (Supplementary Appendix C).

## Discussion

Pre-hospital ECPR studies have been encouraging, with promising survival rates and neurological outcomes.^12^, ^33^ Initial cost effectiveness studies have been favourable but are modelling based.^9^, ^10^, ^11^ To our knowledge, our study is the first PH-ECPR cost effectiveness analysis to include in-trial cost and outcome data. Importantly all known costs to train, start and implement a pre-hospital ECPR service were included.

We have previously reported modelling data showing that ECPR is potentially cost-effective compared to other accepted medical interventions.^9^ This is known also supported by updated data from our current PH-ECPR study albeit with significant variability owing to the small sample size. Moreover, this finding is highly dependent on factors including: the relatively low number of ECPR cases and therefore time the team spends on ECPR cases, neurologically intact survival rates and organ donation.

PH-ECPR has been shown to increase access to ECPR, and reduce times to initiation of ECMO support,^3^, ^34^ which is a consistently significant prognostic factor.^35^ We have previously estimated that the burden of potential PH-ECPR cases in Sydney would be 100–150 PH-ECPR patients per year.^27^ However, our actual patient recruitment has been substantially less than this, primarily because of the limited availability of PH-ECPR expertise (three days a week, during business hours only) and most eligible OHCA cases occur outside of these hours.^36^ This low frequency of PH-ECPR events has several implications for cost effectiveness and budget variability assessment. First, the proportion of time (and cost) spent on PH-ECPR by our team was lower than initially estimated in our previous paper (i.e. <15% actual vs 25% estimated). Therefore, the overall cost effectiveness of team is predominantly influenced by the other non-ECPR work the team is completing. The “sunk cost” of having pre-hospital physicians attending non-ECPR cases was not examined in this paper, however their use has been shown to be cost-effective in another study of high acuity emergencies.^37^ Our pre-hospital clinicians in this trial were already employed to provide other emergency care, hence PH-ECPR was an extension of their existing roles, which is important for both scalability and sustainability. By contrast, a PH-ECPR team solely employed for this role and no other task would not likely be cost-effective. The relatively low frequency of ECPR cases also led to a lower than anticipated budgetary impact.

Our neurologically intact survival rate (∼31%) was consistent with other studies.^38^, ^39^ The favourable outcomes are likely influenced by strict inclusion criteria, which has been shown to affect outcomes^40^ and cost effectiveness. In addition, the impact of increased rates of organ donation from PH-ECPR patients on cost-effectiveness is substantial.^41^, ^42^, ^43^, ^44^ Kidneys, livers, lungs, and other organs may be donated from ECPR and it has been demonstrated in observational studies, and confirmed in a recent systematic review,^45^ that ECPR may provide an average of over two organs per donor,^46^, ^47^, ^48^, ^39^ higher than our rate thus far. Even a small increase in organ donation will substantially improve an interventions cost effectiveness^44^ as was exemplified in the PARAMEDIC 2 trial^49^ when the benefits on organ donation were included the use of adrenaline for OHCA became cost effective.^50^ Our zero-donation sensitivity analysis showed that removing donation related benefits increased PH-ECPR costs to $45,025 /QALY. However, this was still within accepted cost-effectiveness thresholds. Although never the primary reason for ECPR, organ donation through PH-ECPR should be continued to be reported given the substantial societal benefits^51^ of donation.

All survivors reported good quality of life at follow up, and sixty percent of PRECARE survivors returned, or planned to return to work. The remainder were already retired from work or returned to overseas. These results have significant societal and economic benefits. Return to work rates of approximately 50% has been previously reported in ECPR survivors, of whom 80% remained in sustained employment.^52^ Whilst not formally assessed in our study, this would likely have increased the cost-effectiveness of PH-ECPR, as this metric has been shown to increase the overall cost-effectiveness of OHCA interventions.^53^

In our study, we also examined other models of PH-ECPR service provision to inform future planning (Supplementary Appendices D–F). Whilst further operational feasibility assessment is required, all the service models examined were likely to be cost-effective. While PH-ECPR could be provided by hospital-based teams,^54^ this model of care does not provide the same level of population coverage as mobile pre-hospital teams. However, all ECPR models of care entail significant operational and scalability challenges there are outside the scope of this study.

Lastly, the recent ILCOR 2025 update recommended that prehospital critical-care teams (physician-led) attend adults with non-traumatic OHCA within EMS systems with sufficient resource infrastructure albeit with a weak recommendation and low certainty of evidence.^55^ Cost effectiveness analysis such as this will help improve the certainty of evidence and strength of recommendation.

### Limitations

The small sample size introduced substantial variability into the modelling and may limit generalizability of the findings to a broader population. Larger comparative studies are required. The study was restricted to the NSW health system, and therefore many not be generalisable to other health systems. Patient outcomes were tracked up to hospital discharge, without evaluation of subsequent healthcare expenditures related to the initial cardiac arrest. The estimation of HRQoL via CPC scores is based on broad functional outcome categories and may lack the sensitivity provided by comprehensive HRQoL instruments with extended follow-up periods. It was assumed that all patients within the cohort would not have survived without ECPR at 60 min. Although survival with conventional CPR at this time point is possible, large cohorts have found the number to be negligible. The budget impact of a potential fully operational 24/7 or 12/7 PH-ECPR service would need to include the cost of most of the time (∼85%) the prehospital team are undertaking other clinical activity in addition to ECPR and would be impacted by various additional staff costs related to evening and weekend working. Evaluation of the cost effectiveness of a mobile cardiac arrest team involved in the management of non-ECPR cases was outside the scope of this analysis. In contrast to our findings, hospital-based initiation of ECPR support for OHCA has not been shown to be cost-effective.^6^ This is likely due to the lower reported survival rate (20%) in this study. While PH-ECPR may potentially improve survival compared to both hospital-based ECPR (due to faster implementation) and conventional resuscitation, further comparative studies on its clinical utility and cost-effectiveness are required. Our cost apportion to ECPR in the base case (15%) was higher than calculated time on ECPR (6%). This was done to ensure conservative assessment of cost effectiveness (i.e. more costs to PH-ECPR) and a lower percentage of cost apportion would only increase the cost effectiveness of the service. We also deliberately assessed only the impact of kidney donation on the cost effectiveness, which biases the cost effectiveness against PH-ECPR. However, our goal was to be as conservative as possible as to the potential benefit and cost effectiveness of PH-ECPR. Furthermore, kidney donation has very robust health economic evidence that could be utilised. Data Incorporation of any other donated organs would have substantially improved the cost effectiveness and should be assessed in future ECPR studies.

## Conclusion

Cost effectiveness analysis indicates that PH-ECPR in Sydney is likely to be cost effective assuming the team is completing other tasks when not performing ECPR. Survival rates, organ donation, and the team's ability to perform other high acuity clinical tasks are key factors influencing cost effectiveness.

## CRediT authorship contribution statement

**Fredrick Zmudzki:** Writing – review & editing, Writing – original draft, Project administration, Methodology, Investigation, Formal analysis, Data curation. **Brian Burns:** Writing – review & editing, Data curation, Conceptualization. **Natalie Kruit:** Writing – review & editing, Investigation. **Changle Song:** Writing – review & editing. **Emily Moylan:** Writing – review & editing. **Paul Forrest:** Writing – review & editing, Supervision. **Miles Greenberg:** Writing – review & editing, Data curation. **Thomas Evens:** Writing – review & editing, Data curation. **Anthony Keech:** Writing – review & editing, Supervision. **Mark Dennis:** Writing – review & editing, Writing – original draft, Methodology, Investigation, Funding acquisition, Formal analysis, Data curation, Conceptualization.

## Funding

This project was supported by the New South Wales, Ministry of Health Senior and Early-Mid Career Researcher Grants 2023. MD is supported by funding from the National Heart Foundation of Australia and The University of Sydney (M.D.).

## Declaration of competing interest

The authors declare no conflict of interest.
