# Supplementary material for: Cost effectiveness of pre-hospital extracorporeal cardiopulmonary resuscitation for out-of-hospital cardiac arrest – An analysis of the PRECARE study
Source: Resusc Plus. 2026 Jan 22;28:101242. doi: 10.1016/j.resplu.2026.101242 (PMC12906093; doi:10.1016/j.resplu.2026.101242)
Supplement: Supplementary Data 1 [file mmc1.docx]

**Supplementary Appendices**

A Complete Markov model structure

B Parameters used in economic Markov modelling including median and IQR costs

C Supplementary cost effectiveness results, cost effectiveness plane and organ donation sensitivity analysis

D PH-ECPR Alternative staffing scenario

E PH-ECPR estimated cost effectiveness including alternative staffing scenario

F PH-ECPR model scenario assumptions

**Supplementary material – A Complete Markov model structure**

Supplementary figure S1: PH-ECPR Markov model framework - CCPR, IH-ECPR plus 4 PH-ECPR strategies

**
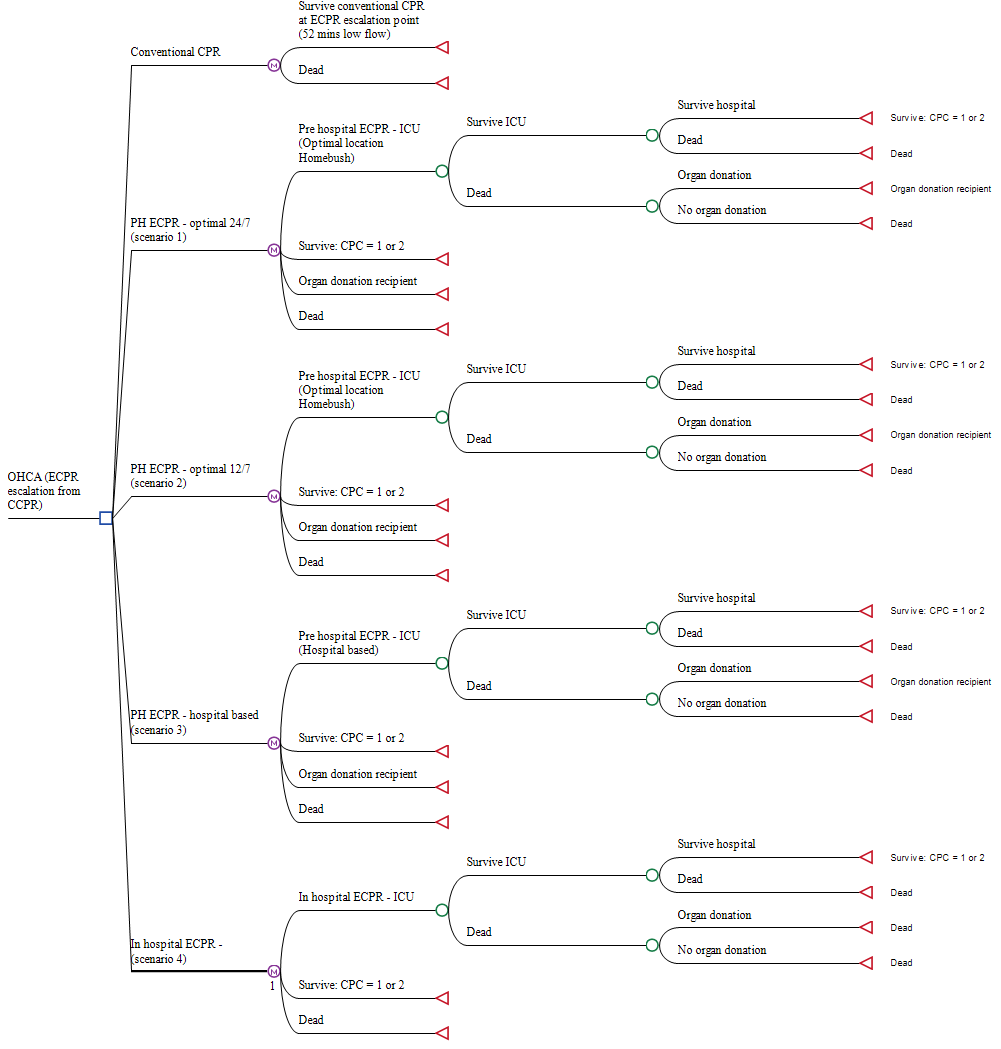
**

**Validation of cost inputs and model mapping**
Hospital‑episode costs were mapped directly from Table 3 to Markov model distributions, including PH‑ECPR team cost (AUD 8,607), ECMO support, ICU and ward length‑of‑stay, cardiac interventions and complications. These were implemented via gamma distributions with means and standard deviations taken from Table 3. The combined hospital‑episode block (mean 85,853 AUD) plus PH‑team cost (8,607 AUD) provides the expected total mean (94,460 AUD). A one‑off transplant cost for kidney donation (105,965 AUD) was applied among non‑survivors producing an expected initial increment of 6,622 AUD in line with the donor fraction of 6.25%. The donation probability used in the model (6.25%) reflects the proportion of PRECARE patients who died in hospital and became successful kidney donors (1 donor of 16 patients). This aligns with published ECPR donor rates of ~7.5% in large observational cohorts, where approximately 1.5 kidneys are transplanted per donor with ~94% one‑year graft survival.

Post discharge donor recipient flows incorporate published net annual cost offsets (dialysis avoided minus transplant maintenance) and a QALY uplift of 0.12 per surviving graft year, consistent with reported kidney graft survival (94.1%) and the average 1.5 grafts per donor seen in ECPR donation series (1). All post discharge costs and utilities occur in annual cycles (years 1–10) with 3.5% discounting.

**Supplementary material - B**

Supplementary Table S1. Descriptive medians and interquartile ranges (IQR) for acute‑care cost components

Table S1a. Percentile summary — survivors vs non‑survivors

| Cost component (AUD) | Survivors (n=5) P25 | P50 (Median) | P75 | Non‑survivors (n=11) P25 | P50 (Median) | P75 |
| --- | --- | --- | --- | --- | --- | --- |
| ECMO support | 18,920 | 23,400 | 29,880 | 14,750 | 20,900 | 33,600 |
| ICU length‑of‑stay cost | 54,200 | 71,900 | 126,500 | 9,300 | 18,770 | 32,950 |
| Ward length‑of‑stay cost | 11,800 | 17,950 | 31,300 | — | — | — |
| Cardiac interventions | 3,900 | 5,200 | 6,700 | 3,200 | 4,900 | 7,200 |
| Complications | 950 | 2,300 | 4,950 | 0 | 950 | 2,900 |
| Total hospital cost | **103,400** | **141,700** | **189,900** | **31,600** | **57,900** | **92,500** |

Table S1b. Percentile summary — Total cohort

| Cost component (AUD) | Total cohort (n=16) P25 | P50 (Median) | P75 |
| --- | --- | --- | --- |
| ECMO support | 17,900 | 23,000 | 31,400 |
| ICU length‑of‑stay cost | 18,200 | 34,900 | 78,500 |
| Ward length‑of‑stay cost | 4,100 | 11,250 | 22,900 |
| Cardiac interventions | 3,600 | 4,700 | 6,900 |
| Complications | 400 | 1,700 | 3,500 |
| Total hospital cost | **58,900** | **91,400** | **148,200** |

Notes

1. Percentile summaries reflect descriptive distributional spread. Economic modelling uses mean costs in line with health economic methodological guidance (Briggs & Gray, BMJ 1999; CHEERS 2022).
2. Ward length of stay cost is blank for non‑survivors (no ward admission).
3. All costs presented in 2025 Australian dollars (AUD).

Supplementary table S2: Parameters used in economic Markov modelling

| **Parameter** | **Unit** | **Value** | **Distribution** | **Range** | **Source** |
| --- | --- | --- | --- | --- | --- |
| **PH-ECPR Costs** |  |  |  |  |  |
| PH-ECPR 100% | Per patient | $57,383 | Gamma | ± $5,738 | 1: NSW Ambulance |
| PH-ECPR 50% | Per patient | $28,692 | Gamma | ± $2,869 | 2: NSW Ambulance |
| PH-ECPR 25% | Per patient | $14,346 | Gamma | ± $1,436 | 3: NSW Ambulance |
| PH-ECPR 15% | Per patient | $8,607 | Gamma | ± $860 | 4: NSW Ambulance |
| Hospital based PH-ECPR | Per patient | $5,416 | Gamma | ± $5,41 | 5: Calculated |
| **Hospital costs** |  |  |  |  |  |
| ECMO support | Per patient | $25,513 | Gamma | ± $ 29,476 | 6: PRECARE trial data |
| Hospital ICU LOS | Per patient | $45,133 | Gamma | ± $52,537 | 6: PRECARE trial data |
| Hospital ward LOS | Per patient | $ 6,914 | Gamma | ± $13,707 | 6: PRECARE trial data |
| Procedures | Per patient | $ 5,783 | Gamma | ± $4,071 | 6: PRECARE trial data |
| Complications | Per patient | $ 2,510 | Gamma | ± $ 3,666 | 6: PRECARE trial data |
| **Probabilities** |  |  |  |  |  |
| PH-ECPR survival | Probability | 31.25% | n/a | Constant | 6: PRECARE trial data |
| IH-ECPR survival | Probability | 16.56% | n/a | Constant | 7: PH-ECPR mapping (2) |
| HB-ECPR survival | Probability | 23.38% | n/a | Constant | 8: PH-ECPR mapping (2) |

Notes: Figures reported in 2024 AUD. ± = standard deviation assumes 10%, LOS = length of stay, IH = in hospital, HB = hospital based

Sources:

1. NSW Ambulance, PH-ECPR PRECARE trial annualised team costs. Average cost per patient assumes 100 PH-ECPR patients per annum. PH-ECPR total service assumes 100% team allocation for ECPR patient responses, turn backs and related activity.
2. NSW Ambulance, PH-ECPR PRECARE trial annualised team costs. Average cost per patient assumes 100 PH-ECPR patients per annum. PH-ECPR service assumes 50% team allocation for ECPR patient responses, turn backs and related activity.
3. NSW Ambulance, PH-ECPR PRECARE trial annualised team costs. Average cost per patient assumes 100 PH-ECPR patients per annum. PH-ECPR service assumes 25% team allocation for ECPR patient responses, turn backs and related activity.
4. NSW Ambulance, PH-ECPR PRECARE trial annualised team costs. Average cost per patient assumes 100 PH-ECPR patients per annum. PH-ECPR service assumes 15% team allocation for ECPR patient responses, turn backs and related activity.
5. NSW Ambulance team composition and cost, hospital based assumed PH-ECPR cases = 100 per year, response turn backs based on PRECARE rate of ~ 1.8 per PH-ECPR eligible case, implicit allocation for pre hospital service management and training based on table 1, ECMO car cost included from table 1. Turn backs assumed 35% day and 65% night / weekend proportions (3).
6. NSW Ambulance, PH-ECPR PRECARE trial annualized team costs, PRECARE patient data.
7. Existing in hospital ECPR based on assumed paramedic on scene time of 27 minutes and cannulation time of 15 minutes (2).
8. Hospital based PH-ECPR on 1 alternative mobile team + 5 current fixed in hospital ECPR hospitals, including assumed 2 minute additional delay from first ambulance arrival to dispatching the mobile team and cannulation time of 22 minutes (2).

**Utility assumptions**

Utilities were derived from CPC categories using the conservative approach employed in our previous ECPR cost‑effectiveness model (4). CPC 1 was assigned a mean utility of 0.85 (SD 0.05), reflecting that survivors with CPC 1 typically demonstrate HUI values above 0.8. CPC 2 was conservatively assigned a utility of 0.40, representing the lower end of the range observed in functional outcome studies (5). CPC‑based utility bridging provides broad category estimates rather than granular health‑state values; therefore, sensitivity analyses were used to address uncertainty in these inputs.

**Supplementary Appendix C**

**Supplementary Table S3: PH-ECPR supplementary cost effectiveness results**

|  | **Cost** | | **Effectiveness** | | **Cost per QALY** |
| --- | --- | --- | --- | --- | --- |
| **Base case and scenarios** | **Total**  **cost** | **Incremental cost** | **QALYs** | **Incremental QALYs** |  |
| **Optimal location – Olympic Park Ambulance base – 24 hour 7 days per week** | | | | | |
| 1a) Base case | $77,469 | $77,469 | 2.3 | 2.3 | $33,990 |
| 1b) Base case (core staffing) |  |  |  |  |  |
| 1c) Base case (inclusion age increased to 75) |  |  |  |  |  |
| **Optimal location – Olympic Park Ambulance base – 12 hour 7 days per week** | | | | | |
| 2a) PRECARE 12/7 A/H expedite | $73,829 | $73,829 | 1.8 | 1.8 | $40,035 |
| 2b) PRECARE 12/7 A/H hospital |  |  |  |  |  |
| **Hospital based – RPA or Westmead hospitals** | | | | | |
| 3a) Hospital based 24/7 | $74,328 | $74,328 | 1.7 | 1.7 | $42,942 |
| 3b) Hospital based 12/7 |  |  |  |  |  |
| **Existing in hospital ECPR (no prehospital service)** | | | | | |
| 4) In hospital ECPR | $86,093 | $86,093 | 1.2 | 1.2 | $73,361 |

Source: NSW Ambulance, PH-ECPR PRECARE trial annualized team costs, Table 2. PRECARE patient data. PH-ECPR base case assumes 15% team allocation for ECPR patient responses, turn backs and related activity. Figures reported in 2025 AUD. Notes:

**Prehospital ECPR 5% team cost allocation sensitivity analysis**

If the estimated PH-ECPR team cost is based on 5% rather than the base case 15% the average cost per PH-ECPR patient is $2,869 (based on assumed 100 patients, $57,383 from Table 2 in manuscript *0,05).
Reducing the allocated cost per PH-ECPR patient by this $5,738 (base case $8,607 - $2,869) reduces the estimated cost per QALY to $31,638:

**Results:**
• Total PH‑ECPR cost = 71,920 AUD
• Incremental QALYs = 2.273
• ICER = 31,638 AUD per QALY

**Supplementary figure S2: PH-ECPR cost‑effectiveness plane Incremental cost effectiveness scatterplot, PH ECPR - 24/7 (scenario 1) vs. escalation point from conventional CPR**


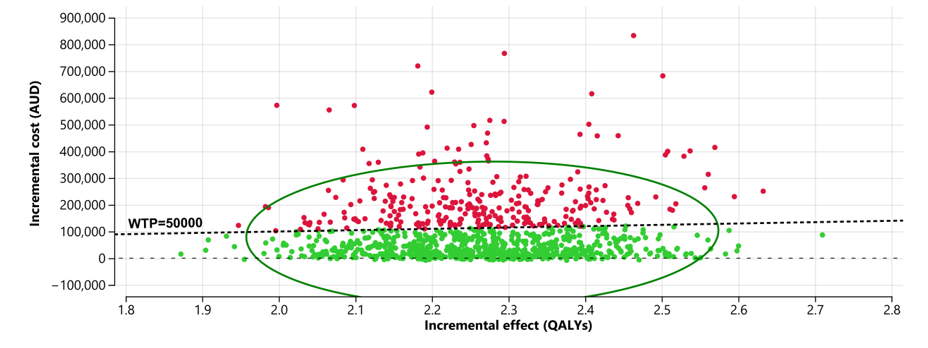


The distribution of PH‑ECPR bootstrap estimates shows that most iterations fall in the north‑east quadrant, indicating the higher costs and higher QALYs relative to the conventional CPR escalation point. The green points represent iterations below the AUD 50,000 willingness‑to‑pay (WTP) threshold, while red points represent iterations above the threshold.

The estimated green cost‑effective region reflects the large variation in incremental costs driven by the small sample size, but with bounded incremental QALYs around 2.0 to 2.6. The lower boundary at zero incremental cost is expected because negative incremental costs cannot occur, i.e. PH-ECPR costs cannot fall below the cost of conventional CPR.

The 95% confidence ellipse (green outline) demonstrates that while most simulations cluster within a plausible cost range, a minority of high‑cost outlier iterations occur above the WTP threshold, consistent with uncertainty from small patient numbers and variable PH-ECPR patient hospital LOS.

**Organ donation sensitivity analysis**

Organ‑donation probability was set to zero, and all transplant costs, dialysis offsets and QALY benefits were removed from the base case

**Results:**
• Total PH‑ECPR cost = 99,518 AUD
• Incremental cost vs CCPR = 5,841 AUD
• Incremental QALYs = 1.039
• ICER = 45,025 AUD per QALY

Removing organ‑donation effects increases the cost per QALY relative to the base case. However, PH‑ECPR remains within accepted cost‑effectiveness thresholds, consistent with overall model conclusions.

**Supplementary Appendix D – PH-ECPR Alternative staffing scenario**

The alternative staffing scenario excludes the senior staff specialist ECPR clinical lead, assumed one of the staff specialists is lead while on duty. Excludes the dedicated paramedic specialist OHCA clinician on dispatch, assumed PH-ECPR calls are managed through established NSWA dispatchers. And excludes separate paramedic educator. Excluded staffing shaded dark grey compared to table 2 in manuscript.

Supplementary table S4: Prehospital ECPR alternative annual service cost and PH-ECPR team cost per patient

| **Clinical role** | **Hours per week** | **Cost per week** | **Cost per**  **annum** |
| --- | --- | --- | --- |
| Senior Staff Specialist 1 Cannulator | 168 | $37,338 | $1,941,576 |
| Senior Staff Specialist 2 Cannulator | 168 | $37,338 | $1,941,576 |
| Senior Staff Specialist - ECPR Clinical Lead |  |  |  |
| Critical Care Paramedic - Perfusionist | 168 | $9,878 | $513,677 |
| Paramedic Specialist - OHCA Clinician (Dispatch) |  |  |  |
| Paramedic Educator - Perfusionist |  |  |  |
| Total wages for 24 hours 7 days per week | | $84,554 | $4,396,829 |
| Staff Specialist training | | | $120,015 |
| Porcine lab coordination | | | $21,000 |
| Paramedic training (4 weeks) | | |  |
| Senior Staff Specialist - ECPR Clinical Lead | | | $33,782 |
| Critical Care Paramedic - Perfusionist | | | $17,875 |
| Paramedic Educator | | |  |
| PH-ECPR car lease and running cost per annum | | | $50,000 |
| PH-ECPR total annual service cost | | | $4,639,501 |
| Average cost per patient (assumed 100 patients / year) | | | $46,395 |
| Estimated average cost per patient by assumed % PH-ECPR activity | | | |
| 50% PH-ECPR (50% other advanced critical care interventions) | | | $23,198 |
| 25% PH-ECPR (75% other advanced critical care interventions) | | | $11,599 |
| 15% PH-ECPR (85% other advanced critical care interventions) | | | $6,959 |

Source: NSW Ambulance, PH-ECPR PRECARE trial annualized team costs. Average cost per patient assumes 100 PH-ECPR patients per annum. PH-ECPR base case assumes 15% team allocation for ECPR patient responses, turn backs and related activity. Figures reported in 2025 AUD.

**Supplementary Appendix E –** **PH-ECPR estimated cost effectiveness including alternative staffing scenario**

The green curves indicate the estimated increased cost effectiveness at each assumed level of assumed ECPR allocation, based on potentially reduced staffing scenario in Appendix E above.

Supplementary figure S3: PH-ECPR estimated cost effectiveness per patient by strategy


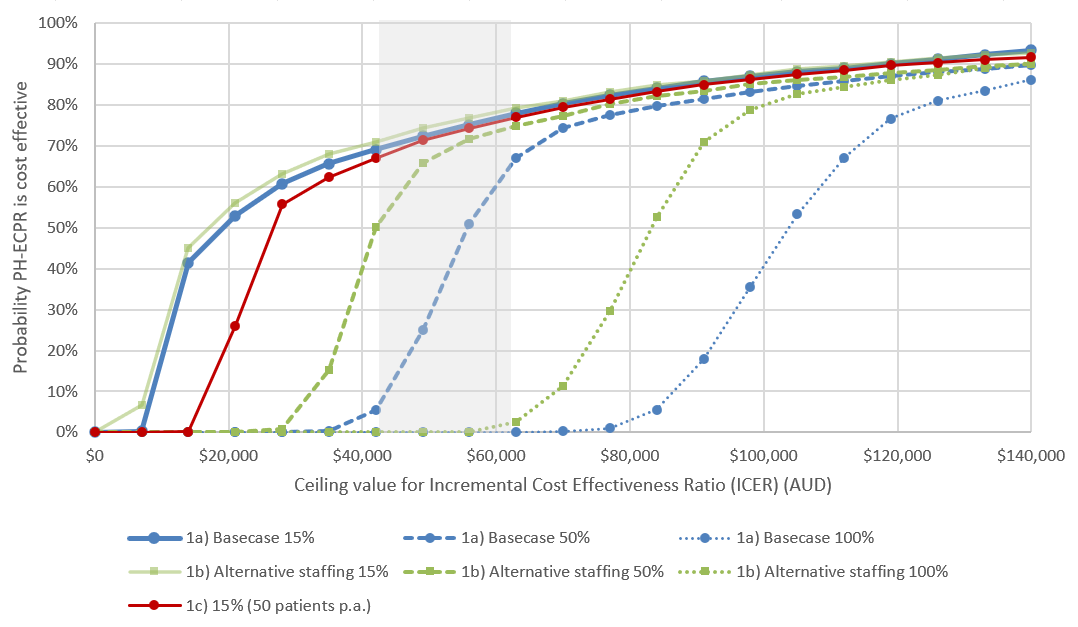


Source: PH-ECPR base case assumes 15% team allocation for ECPR patient responses, turn backs and related activity. Figures reported in 2025 AUD. Notes: The cost effectiveness thresholds are indicated by the shaded segment based on United Kingdom NICE guidance of GBP 20,000 to 30,000 converted at 2.09 AUD = AUD 41,800 to 62,700

**Supplementary Appendix F**

**Supplementary table S5: PH-ECPR model scenario assumptions**

|  | PH-ECPR service model scenario | Hours | PH Patients | Survival^^[[1]](#footnote-1)^^ | PH Team cost | Training | PH-ECPR % allocation | Comments |
| --- | --- | --- | --- | --- | --- | --- | --- | --- |
| Optimal location – Olympic Park Ambulance base – 24 hour 7 days per week | | | | | | | | |
| 1a | Base case - Optimal location 24/7 ^^[[2]](#footnote-2)^^ | 24/7 | 100 (50 – 150) | PRECARE 5/16 =31.3% | $5.7 million | $120,015 | 15% | Full NSWA team:  2 senior staff specialists  Senior staff specialist – ECPR lead  1 paramedic  1 dispatcher  Paramedic educator |
| 1b | Base case - Optimal location 24/7  (Core staffing team) | 24/7 | 100 (50-150) | Assumed 30% | $4.6 million | $120,015 | 15% | 2 senior staff specialists  1 paramedic |
| 1c | Base case - Optimal location 24/7 (Age protocol from 70 to 75 years) | 24/7 | 200 | Assumed 30% | $5.7 million | $120,015 | 15% | Full NSWA team 1a |
| Optimal location – Olympic Park Ambulance base – 12 hour 7 days per week | | | | | | | | |
| 2a | NSWA 12/7 Optimal location. After hours ECPR eligible patients expedited to Westmead, RPA or SVH for hospital based ECPR ^^[[3]](#footnote-3)^^ | 12/7  9am – 9pm | 50 in hours  +20 after hours | Assumed 30% | Assumed 50% 1a  plus $2,984 ^^[[4]](#footnote-4)^^ per patient  $2.9 million | Current trained staff | 15% | Full NSWA full team 50% 1a  After hours includes on call Cardiologist, ICU and anaesthetic VMO + 2 nurses |
| 2b | NSWA 12/7 Homebush  After hours ECPR eligible patients covered by hospital ECMO specialist ^^[[5]](#footnote-5)^^ | 12/7  9am – 9pm | 50 in hours  +20 after hours | Assumed 30% | Assumed 50% 1a  plus $2,984 per patient  $2.9 million | Current trained staff | 15% | Full NSWA full team 50% 1a  After hours includes on call Cardiologist, ICU and anaesthetic VMO + 2 |
| Hospital based – RPA or Westmead hospitals | | | | | | | | |
| 3a | Hospital based 24/7 ^^[[6]](#footnote-6)^^  (Melbourne model) | 24/7 | Assumed  100 (50-150) | Assumed 30% | On duty staff  (on call after hours $2,984 per patient | Current trained hospital staff | n/a | On duty ECMO specialist + registrar + paramedic (on call after hours). Assumes immediate response with turn backs |
| 3b | Hospital based 12/7  (Melbourne model) | 12/7 | Assumed 50  (in hours) | Assumed 30% | On duty staff | Current trained hospital staff | n/a | On duty ECMO specialist + registrar + paramedic (on call after hours). Assumes immediate response with turn backs |
| Existing in hospital ECPR  (no prehospital service) | | Hours | In hospital patients | Survival | Hospital team cost | Hospital training | PHECPR % allocation |  |
| 4 | In hospital ECPR | 12/5  (Monday - Friday) | Assumed  15 (in hours) | 16.6% Song | On duty staff | Current trained hospital staff | n/a | Currently operating service |

**References**

1. Raphalen JH, Soumagnac T, Blanot S, Bougouin W, Bourdiault A, Vimpere D, et al. Kidneys recovered from brain dead cardiac arrest patients resuscitated with ECPR show similar one-year graft survival compared to other donors. Resuscitation. 2023;190:109883.

2. Song C, Dennis M, Burns B, Dyson S, Forrest P, Ramanan M, et al. Improving access to extracorporeal membrane oxygenation for out of hospital cardiac arrest: pre-hospital ECPR and alternate delivery strategies. Scandinavian Journal of Trauma, Resuscitation and Emergency Medicine. 2022;30(1):77.

3. Zmudzki F, Burns B, Kruit N, Song C, Moylan E, Vachharajani H, et al. Pre-hospital ECPR cost analysis and cost effectiveness modelling study. Resuscitation. 2025:110488.

4. Dennis M, Zmudzki F, Burns B, Scott S, Gattas D, Reynolds C, et al. Cost effectiveness and quality of life analysis of extracorporeal cardiopulmonary resuscitation (ECPR) for refractory cardiac arrest. Resuscitation. 2019.

5. Stiell IG, Nesbitt LP, Nichol G, Maloney J, Dreyer J, Beaudoin T, et al. Comparison of the Cerebral Performance Category score and the Health Utilities Index for survivors of cardiac arrest. Ann Emerg Med. 2009;53(2):241–8.

1. Assume 30% survival for all models, PRECARE actual survival data used [↑](#footnote-ref-1)
2. 24/7 days a week, PRECARE trial annualised [↑](#footnote-ref-2)
3. With direct phone call to ECMO team (i.e. after hours there is no pre-hospital ECPR service) [↑](#footnote-ref-3)
4. Based on assumed Cardiologist for angiogram (2 hours), 2 nurses for angiogram (2 hours each), ICU staff specialist VMO (4 hours), anaesthetist VMO (3 hours) [↑](#footnote-ref-4)
5. With a car stationed at the hospital and they attend arrests etc when they are on "shift" [↑](#footnote-ref-5)
6. Clinician only responding from hospital, (RPA or Westmead) no NSWA PH-ECPR team [↑](#footnote-ref-6)
